# Perampanel-Induced, New-Onset Thrombocytopenia in a Patient With Refractory Seizures: A Case Report

**DOI:** 10.7759/cureus.37781

**Published:** 2023-04-18

**Authors:** Rakan A Almuhanna, Rabia Muddassir, Murouj Almaghrabi, Gadir Bokhari, Abdulazuiz Al-Ghamdi

**Affiliations:** 1 Neurology, Security Forces Hospital, Makkah, SAU; 2 Internal Medicine, Security Forces Hospital, Makkah, SAU; 3 Medicine and Surgery, College of Medicine, Umm Al-Qura University, Makkah, SAU; 4 Medicine, College of Medicine, Umm Al-Qura University, Makkah, SAU

**Keywords:** adverse effect, hematological complication, thrombocytopenia, fycompa, perampanel

## Abstract

Perampanel (Fycompa) is a glutamate receptor antagonist known to be a safe, effective, and well-tolerated medication; nevertheless, adverse effects are possible. This case report aims to raise the suspicion of perampanel-induced thrombocytopenia and discuss its possible pathways implicated.

Here, we present the case of a 66-year-old female patient with a generalized tonic-clonic seizure initially managed with levetiracetam, valproic acid, and lacosamide; however, the patient continued to have seizures clinically as well as on the electroencephalogram. The patient was initiated on 2 mg of perampanel and reached up to 12 mg within a week, after which the seizure was controlled. Nevertheless, after perampanel initiation, a gradual platelet count reduction was observed. Upon withdrawal of perampanel, the platelet count dramatically improved reaching up to her baseline.

Although perampanel is known to be a safe medication, a hematological complication such as thrombocytopenia is possible. The exact mechanism remains unclear. Further studies are required to understand the association between thrombocytopenia and perampanel to identify high-risk populations and prevent this condition sequentially.

## Introduction

Anti-seizure drugs (ASDs) are frequently used to treat epilepsy and prevent seizure attacks by controlling hypersynchronous activity and hyperexcitability in brain circuits. Recent research has identified the following four major classes of ASD mechanism of action: (i) voltage-gated ion channel modulation; (ii) neurotransmitter release modulation via a presynaptic action; (iii) enhancement of γ-aminobutyric acid-mediated inhibitory neurotransmission; and (iv) attenuation of glutamate-mediated excitatory neurotransmission. In the nervous system, glutamate is known to be the main excitatory neurotransmitter in the human brain. After being released from glutamatergic nerve terminals, it acts on postsynaptic membrane ionotropic receptors, and one of these receptor subtypes is the α-amino-3-hydroxy-5-methyl-4-iso acid (AMPA) glutamate receptor [1].

Perampanel (Fycompa) is a novel, noncompetitive AMPA glutamate receptor antagonist. The Food and Drug Administration (FDA) approved it in October 2012 as an adjunctive treatment for primary generalized tonic-clonic seizures and focal seizures with or without secondary generalization among epilepsy patients older than 12 years [2].

Clinical trials demonstrate that perampanel is a safe, effective, and well-tolerated medication in general; nevertheless, there are a few widespread side effects related to the central nervous system, including dizziness, drowsiness, ataxia, somnolence, headaches, and blurred vision [3,4]. In addition, severe psychiatric and behavioral reactions are possible, including aggression, irritability, and anxiety [1].

Reviewing the literature, studies have yet to identify the relationship between thrombocytopenia and perampanel. Thus, this study aims to raise the suspicion of perampanel-induced thrombocytopenia and discuss possible pathways implicated. In addition, we report the case of a female patient with generalized tonic-clonic seizure who developed thrombocytopenia and had a dramatic improvement in thrombocytopenia upon withdrawing the drug.

## Case presentation

A 66-year-old female patient was a known case of hypertension, diabetes mellites, severe mitral regurgitation, and ischemic stroke with left-sided weakness four years before presentation, followed by left foot below-the-knee amputation two weeks before presentation secondary to gangrene. The patient presented to the emergency department with complaints of dyspnea, productive cough, and low-grade fever for three days. She was admitted as a case of congestive heart failure secondary to mitral regurgitation along with hospital-acquired pneumonia. She was started on diuretics and ceftriaxone 2 g, once daily, for three days.

On the first day of admission, the patient had a generalized tonic-clonic seizure lasting for 10 seconds and was started on intravenous levetiracetam 500 mg twice daily.

Post-seizure examination revealed that the patient was conscious, oriented, and following one-step commands. The patient’s vital signs showed a blood pressure of 134/50 mmHg, a pulse of 70 beats/minute, a temperature of 37.8°C, and oxygen saturation of 93% on 3 L of oxygen. The neurological examination showed no gaze preference, and pupils were bilaterally equally reactive to light and of normal size. She was localizing with the right upper limb. The left upper and lower limb was spastic due to a previous stroke. Reflexes on the left upper limb and lower limb were 3/5.

Furthermore, reflexes in the right upper and lower limbs were 2/5. The neck was supple with no signs of meningeal irritation. The respiratory examination was positive for bilateral basal crepitations and a harsh mid-diastolic murmur. The rest of the systemic examination was unremarkable.

In the following three days, the patient had no seizures. The antibiotic was stopped as shortness of breath was diagnosed due to heart failure rather than aspiration pneumonia.

**Figure 1:**
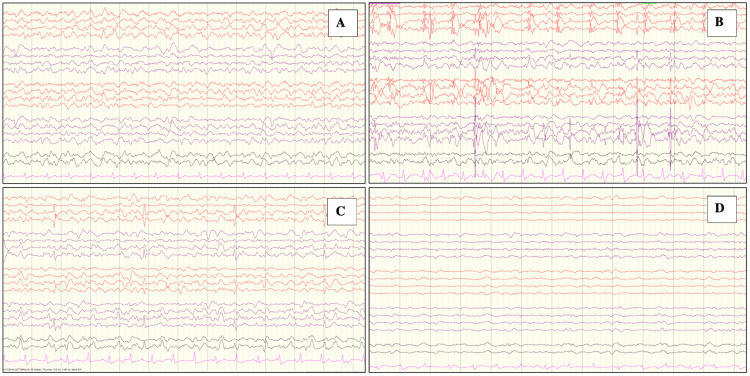
Electroencephalogram activity of the patient during four different episodes. (A) Showing only diffuse slowing with no epileptic form charges. (B) Intensive care unit admission showing status epilepticus. (C) Initial follow-up showing status epilepticus. (D) Post-initiation of perampanel revealing improvement.

On day four, she started to have continuous myoclonic activity localized to the left lower amputated limb. On examination, the patient was conscious, oriented, and following commands with no gaze preference. The electroencephalogram (EEG) showed diffuse slowing with no epileptiform discharges (Figure 1, Panel A).

**Figure 2:**
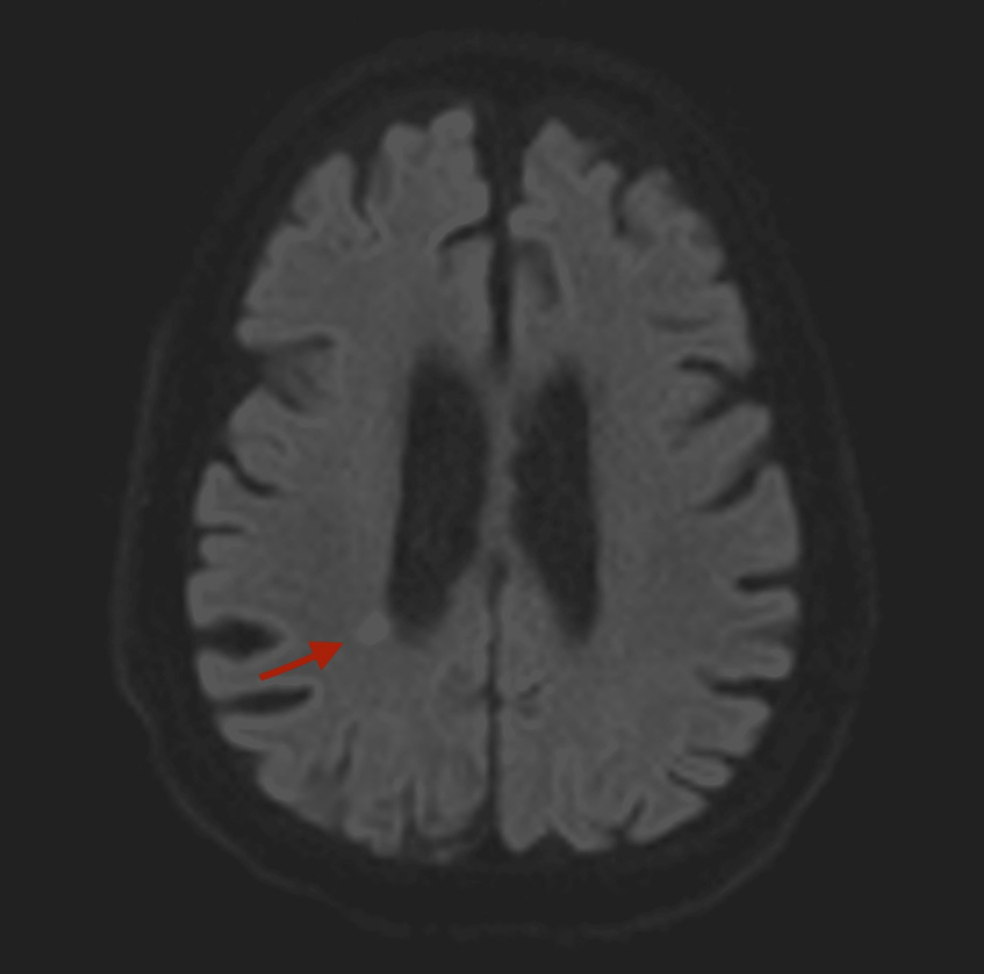
An axial section of magnetic resonance imaging of the brain, diffusion-weighted imaging (DWI) sequence, showing a small, restricted area in the posterior right periventricular partial region likely subacute infarction.

A computerized tomography (CT) scan of the brain was unremarkable. Magnetic resonance imaging (MRI) of the brain, diffusion-weighted image (DWI), showed a small restricted area in the posterior right periventricular parietal lesion, likely subacute infarction (Figure 2). At this time, the movement was diagnosed as non-epileptic activity.

The patient was given a trial of 5 mg midazolam followed by a loading dose of levetiracetam and sodium valproate, decreasing the movement but not aborting it altogether. On day five, the myoclonic movement progressed to the left upper limb involving the left side of the face with head deviation to the left side. On examination, the patient was confused, not following commands with an impaired level of awareness, and was shifted to the intensive care unit (ICU) with an impression of status epilepticus. 

**Figure 3:**
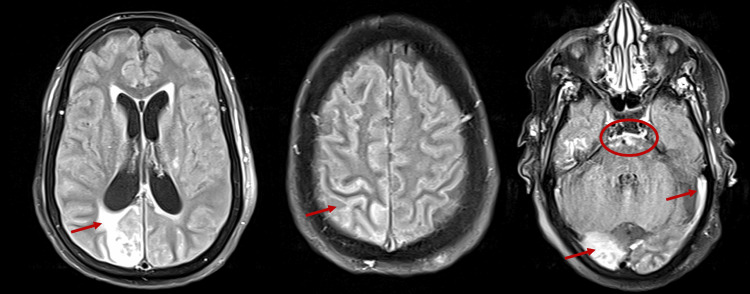
Magnetic resonance imaging (MRI) of the brain with fluid-attenuated inversion recovery sequence. The image a newly developed abnormal signal seen at the right high parietal region that exhibits a hyperintense signal, as well as a newly developed effacement of the pontine cisterns with abnormal signal intensity and abnormal signal of the cerebrospinal fluid seen at the extra-axial spaces.

EEG showed status epilepticus (Figure 1, Panel B). The patient was intubated electively and sedated. An MRI of the brain with contrast showed a newly developed abnormal signal at the right high parietal area suspected of an infectious process (Figure 3).

Laboratory investigation in the ICU revealed a hemoglobin of 10.5 g/dL, total leucocytes of 6.43 fL, and platelet level of 262 × 10^6^/µL (Table 1). Renal function tests were normal. Inflammatory markers revealed an erythrocyte sedimentation rate (ESR) of 80 mm/hour, C-reactive protein (CRP) of 26.87 mg/L, lactate of 321 U/L, and procalcitonin of 0.08 µg/L. The cerebrospinal fluid (CSF) analysis showed 23 leukocytes with a differential of 23% lymphocytes, 13% monocytes, and 64% neutrophils. The CSF glucose was 7.67 mmol/L, and the CSF protein was 494 mg/L. The CSF culture and gram stain were negative. With the suspicion of partially treated meningitis, the patient was started on a high dose of ceftriaxone and vancomycin.

**Table 1:** Laboratory parameters workup of the patient during the four phases of admission. ICU = intensive care unit; MCV = mean corpuscular volume; MCH = mean corpuscular hemoglobin; MPV = mean platelets volume

Parameter	Admission in the ward	1^st^ day of ICU admission	10^th^ day of ICU admission	17^th^ day of ICU admission	Thrombocytopenia phase	Recovery phase	Reference range (units)
Erythrocytes							
Total count	4.13	4.11	4.29	3.34	2.88	3.93	4.20–5.40 (×10^6^/µL)
Hemoglobin	10.7	10.5	11.2	8.9	9.8	11.0	12.0–15.0 (g/dL)
MCV	82.1	86.6	83.7	88.3	91.7	86.8	81.0–96.0 (fL)
MCH	25.9	25.5	26.1	26.6	26.7	28.0	27.0–32.0 (pg)
Leucocytes							
Total count	10.83	6.43	8.57	16.88	13.91	8.00	4.00–11.00 (×10^3^/µL)
Neutrophils	7.67	3.11	6.86	14.47	12.21	5.02	2–7 (×10^3^/µL)
Lymphocytes	1.27	1.79	0.99	1.01	0.47	1.15	1.00–3.00 (×10^3^/µL)
Basophils	0.06	0.06	0.03	0.02	0.01	0.05	0.00–0.20 (×10^3^/µL)
Eosinophils	0.58	0.99	0.00	0.02	0.26	0.15	0.00–0.70 (×10^3^/µL)
Monocytes	1.25	0.49	0.68	1.37	0.95	1.63	0.00–0.90 (×10^3^/µL)
Platelets							
Total count	275	262	210	270	129	177	150–450 (×10^3^/µL)
MPV	10.40	10.10	9.90	10.50	12.10	10.20	7.40–10.40 (fL)

The initial follow-up EEG was still showing status epilepticus (Figure 1, Panel C) despite the full doses of levetiracetam and valproic acid, and hence, lacosamide was introduced. Following the administration of lacosamide, the EEG was still positive for the seizure; however, the follow-up CSF on the seventh day was normal with a negative gram stain and culture. Therefore, perampanel was introduced at a dose of 2 mg on day 14 of ICU and reached up to 12 mg for a week.

After the initiation of perampanel, the EEG improved progressively (Figure 1, Panel D); therefore, sedation was tapered and stopped.

Other investigations, including the autoimmune workup and the infectious workup for syphilis, brucella, and human immunodeficiency virus, were negative. In addition, CSF tuberculosis polymerase chain reaction (PCR) and acid-fast bacilli, fungal culture sensitivity, and herpes simplex virus PCR twice were all negative.

**Figure 4:**
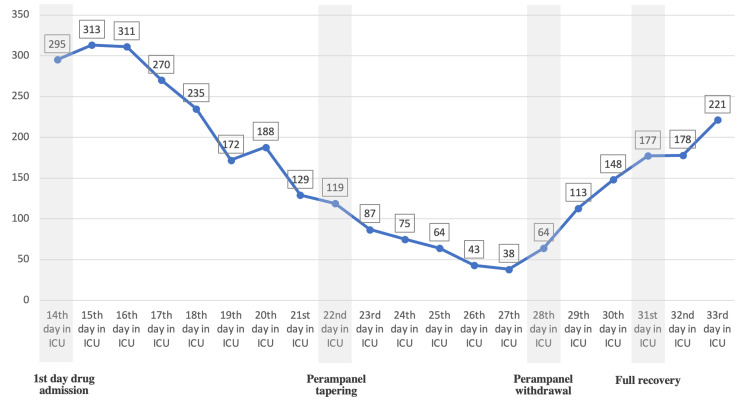
Platelet count monitoring upon receiving perampanel (Fycompa).

After stopping sedation, the patient was still unresponsive; however, there was no clinical seizure, and EEG was negative for any seizure activity. With the diagnosis of drug-induced encephalopathy, levetiracetam was tapered initially, followed by lacosamide. During the ICU stay, the repeated full blood count showed progressive thrombocytopenia (Table 1) that gradually decreased until it reached 38 × 10^6^/µL. Renal parameters were normal, including creatinine 70.60 µmol/L, chloride 108.11 mmol/L, sodium 143.83 mmol/L, potassium 3.30 mmol/L, and urea 17.44 mmol/L. In addition, inflammatory markers (including ESR, CRP, lactate, and procalcitonin) were normal. As no cause of thrombocytopenia was found, with the suspicion of drug-induced thrombocytopenia, a tapering dose of valproic acid was started. However, the patient started to have a clinical seizure, following which valproic acid was resumed at the same initial dose. We started tapering perampanel and stopped it in a period of a week. Following the discontinuation of perampanel, the platelet level started to show improvement and returned to the baseline within three days (Table 1, Figure 4). 

After a follow-up for one month, the patient remained on tracheostomy, opening her eyes spontaneously and not following commands. No more clinical seizures were noted, and the last EEG was negative for seizures.

## Discussion

The current study presents the first case involving thrombocytopenia induced by perampanel. In our patient, a remarkable temporal association was realized between the drug administration and the gradually decreased level of platelets within days (Figure 4). Hence, all possible causes of thrombocytopenia were ruled out, including infections, autoimmune diseases, hematologic disturbance (such as thrombotic thrombocytopenic purpura and disseminated intravascular coagulation), sepsis, and antibiotics.

The old generation of ASDs is associated with hematological disorders, including anemia, neutropenia, thrombocytopenia, and even bone marrow failure. Thus, close monitoring is required when particularly prescribing phenytoin, carbamazepine, or valproate [5]. Regarding sodium valproate, it has been estimated to cause thrombocytopenia in 5-60% of patients [6].

In another study, Alzahrani et al. revealed the case of a 79-year-old female patient who was started on a prophylactic levetiracetam per neurosurgery local protocol and developed pancytopenia soon after drug administration. She recovered after discontinuation of the drug [7]. Furthermore, the study provided a literature review of three cases presenting with various clinical diagnoses, which suggest that levetiracetam was the cause of pancytopenia rather than a side effect of the underlying illnesses.

In general, drug-induced thrombocytopenia can occur by three different main mechanisms, including (i) direct toxic effect; (ii) hapten formation; and (iii) innocent bystander immune response [8]. However, the exact mechanism of ASDs causing thrombocytopenia is not well established; hence, it may result from higher platelet destruction, the development of platelet-destroying autoantibodies, or decreased production due to a direct toxic effect on the bone marrow [9].

Regarding the pharmacokinetics of perampanel, it is a rapidly absorbed medication with a prolonged half-life of approximately 100 hours reached in a median time of 0.5-2.5 hours in fasting status. The drug distribution reaches 95-96% protein-bound [1]. Based on an in-vitro finding, perampanel metabolizes in the liver via oxidation primarily followed by glucuronidation, which is mediated by cytochrome P450 (CYP) 3A4 [2]. The half-life elimination is about 70-110 hours, with a clearance rate of 0.730 L/hour in adult males and 0.605 L/hour in females [10].

Future studies are recommended to address numerous ways by which perampanel causes thrombocytopenia. First, the actual clinical causes of perampanel-associated thrombocytopenia are unclear; for instance, whether it is due to a decrease or inadequate production in bone marrow or destruction in marrow or periphery. Second, the associated parameters with perampanel-induced thrombocytopenia remain unknown, whether it is related to the type of illness, drug interaction, concomitant renal disease, or accompanying another anti-seizure medication. Third, it is unclear how soon hematological abnormalities may appear. Thus, short-term therapy may have a limited effect compared with long-term use of the drug. Lastly, the exact mechanism of perampanel-induced thrombocytopenia needs to be clarified, which will aid in identifying high-risk populations and prevent this adverse event sequentially.

## Conclusions

Although perampanel is known to be a safe medication, a hematological complication such as thrombocytopenia is possible. Close monitoring of the clinical and laboratory parameters among patients who use perampanel is crucial for the prompt detection of adverse effects. We recommend a routine complete blood count check after starting perampanel.

The exact mechanism of perampanel-induced thrombocytopenia remains unclear. Therefore, we highly recommend further studies to understand the clinical causes, related risk factors, association with therapy duration, and the relationship between thrombocytopenia and perampanel to identify high-risk populations and prevent the condition sequentially.
